# Supplementary material for: Design of hierarchical-heterostructure antiferroelectrics for ultrahigh capacitive energy storage
Source: Nat Commun. 2025 Nov 27;16:10668. doi: 10.1038/s41467-025-65694-z (PMC12661050; doi:10.1038/s41467-025-65694-z)
Supplement: Supplementary file 1 — Supplementary Information [file 41467_2025_65694_MOESM1_ESM.pdf]

Supplementary Materials for

Design of hierarchical-heterostructure antiferroelectrics for ultrahigh capacitive energy storage

Liang Chen, Tengfei Hu, He Qi, Huifen Yu, Zhengqian Fu, Shujun Zhang, Jun Chen

Corresponding author. Email: qihe@hainanu.edu.cn (H.Q.); junchen@ustb.edu.cn (J.C.)

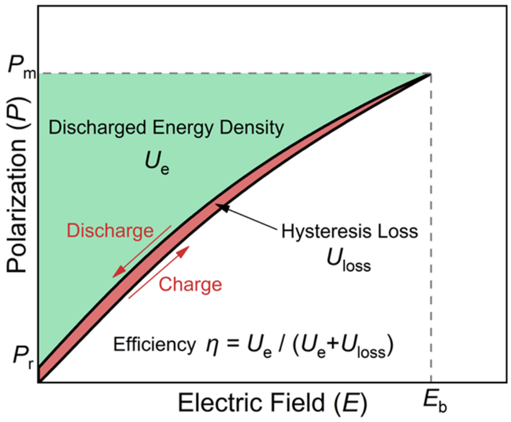

Supplementary Fig. S1 | Schematic diagram of dielectric energy storage.

When an external electric field is applied, dipoles can rotate and establish long-range polarization order, causing electrostatic energy to be charged into dielectrics (polarization rising path in the charging process). The polarization can be released and stored energy can be discharged when removing the electric fields (polarization recovery path in the discharging process). The discharged energy density U_e can be calculated by $U_e = \int_{P_r}^{P_m} E\mathrm{d}P$ and represented by the green area, where P_m and P_r are the maximum polarization and remnant polarization, respectively. The energy storage efficiency η can be computed by $\eta = U_e/(U_e + U_{loss})$, where U_{loss} is the hysteresis or energy loss, which can be signaled by the red area. E_b represents the breakdown electric field. An outstanding energy storage dielectric should have both high U_e and η. Obviously, improving E_b has the ability to improve U_e, but it is powerless to improve η, which generally shows a decreasing trend with increasing electric field. However, controlling polarization evolution behavior (including polarization fluctuation and saturation) under external electric fields can simultaneously improve U_e and η.

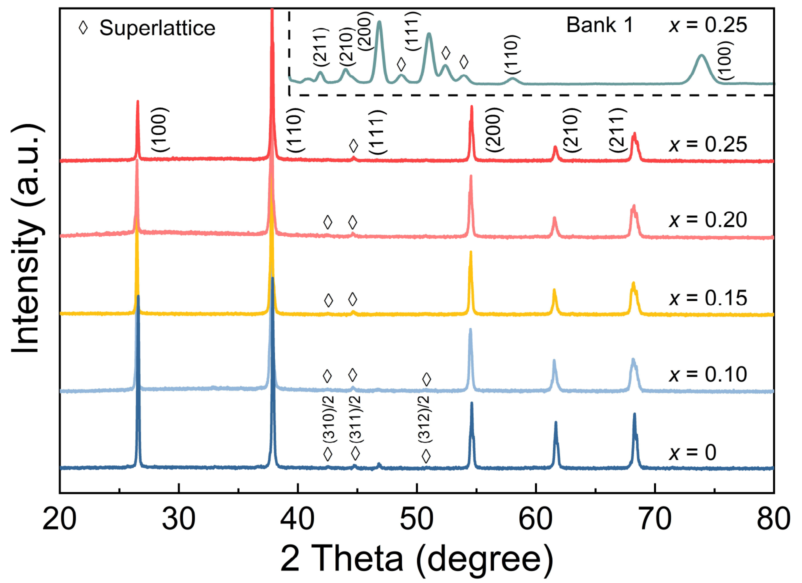

Supplementary Fig. S2 | X-ray diffraction patterns of the NN-CZ-*x*BNT ceramics. The inset is the neutron diffraction pattern of $x = 0.25$.

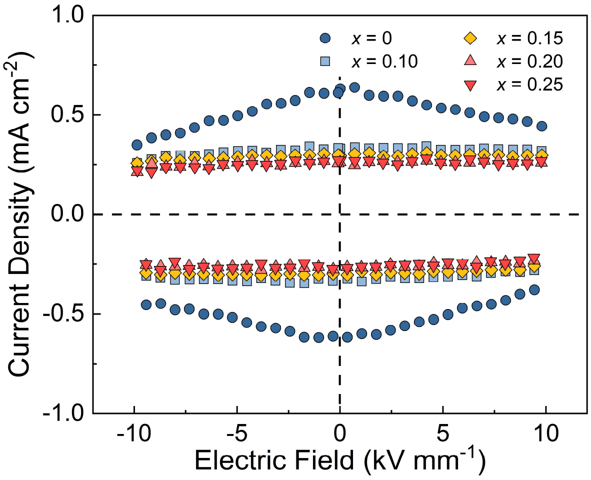

Supplementary Fig. S3 | Current density of the NN-CZ-*x*BNT ceramics at an electric field of 10 kV mm^{-1}.

x = 0 ceramic shows obviously bulging current peaks, which is attributed to the diffused antiferroelectric-ferroelectric phase transition[1,2]. The peaks gradually flatten and decrease with increasing BNT. The flat and small current peaks indicate uniform polarization rotation features with extremely diffused and negligible phase transition behaviors under external electric fields, which can reduce energy consumption[3].

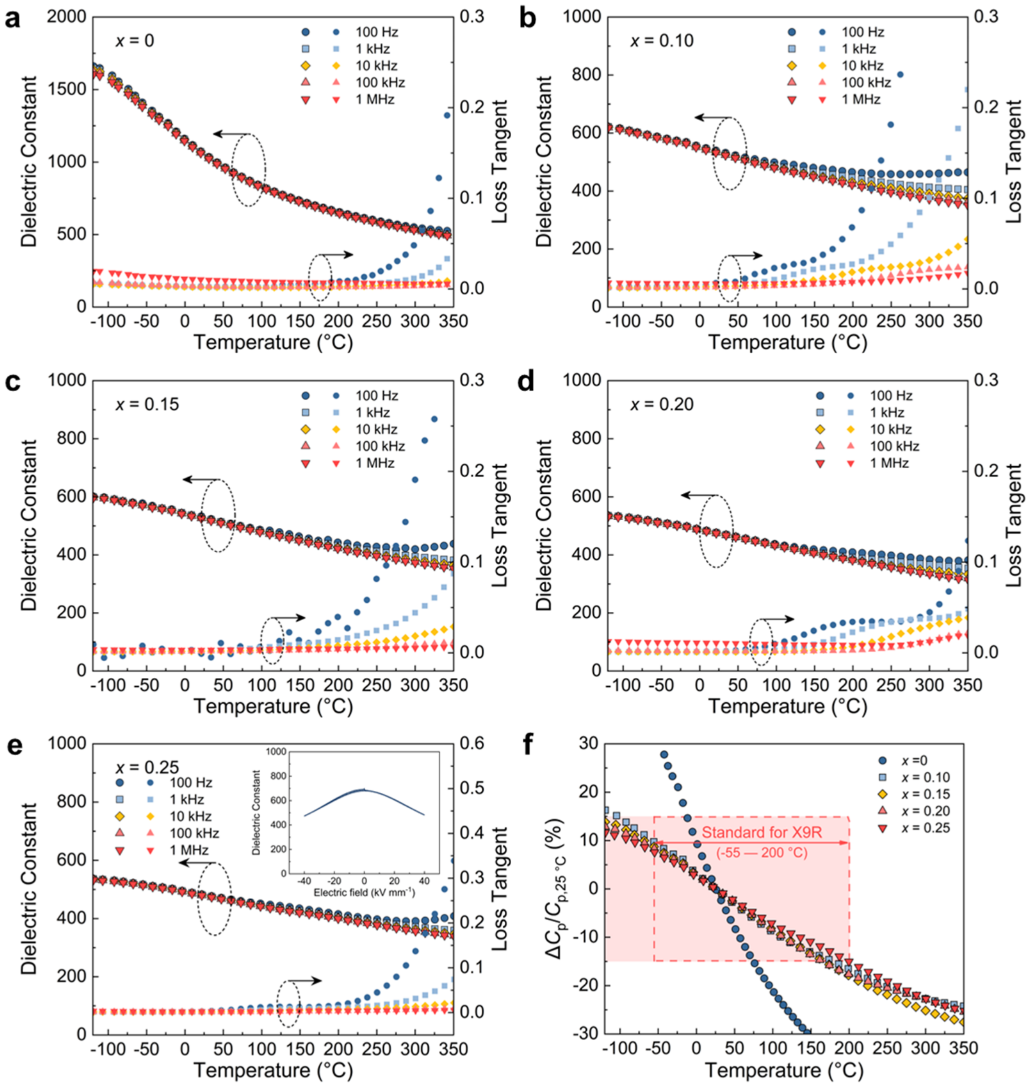

Supplementary Fig. S4 | Dielectric and capacitance properties of the NN-CZ-*x*BNT ceramics. Temperature- and frequency-dependent dielectric spectra for (**a**) $x = 0$, (**b**) $x = 0.10$, (**c**) $x = 0.15$, (**d**) $x = 0.20$, and (**e**) $x = 0.25$ ceramics. The inset in (**e**) is the electric field-dependent dielectric constant for $x = 0.25$ ceramic measured at 10 Hz. (**f**) Capacitance properties ($\Delta C_p/C_{p,25\ °C}$) of the NN-CZ-*x*BNT ceramics at 1 kHz.

The temperature corresponding to maximum dielectric constant (T_m) of all dielectrics cannot be found despite reaching -120 °C, showing that they locate in the superparaelectric region ($T_m \leq T \leq T_B$, Burns temperature) at room temperature, where ultrasmall weakly coupled PNRs exist[2,4]. The decreased room-temperature dielectric constant and weaker temperature dependence of dielectric constant can be discovered in NN-CZ-*x*BNT ceramics with increasing BNT, indicating the enhanced relaxation behavior[5,6]. Moreover, the frequency-independent dielectric constant of all samples can be clearly found at room temperature. As shown in Supplementary Fig. S4e, the dielectric constant of $x = 0.25$ ceramic shows a decreasing trend with increasing electric fields, which is similar to other reported relaxors.

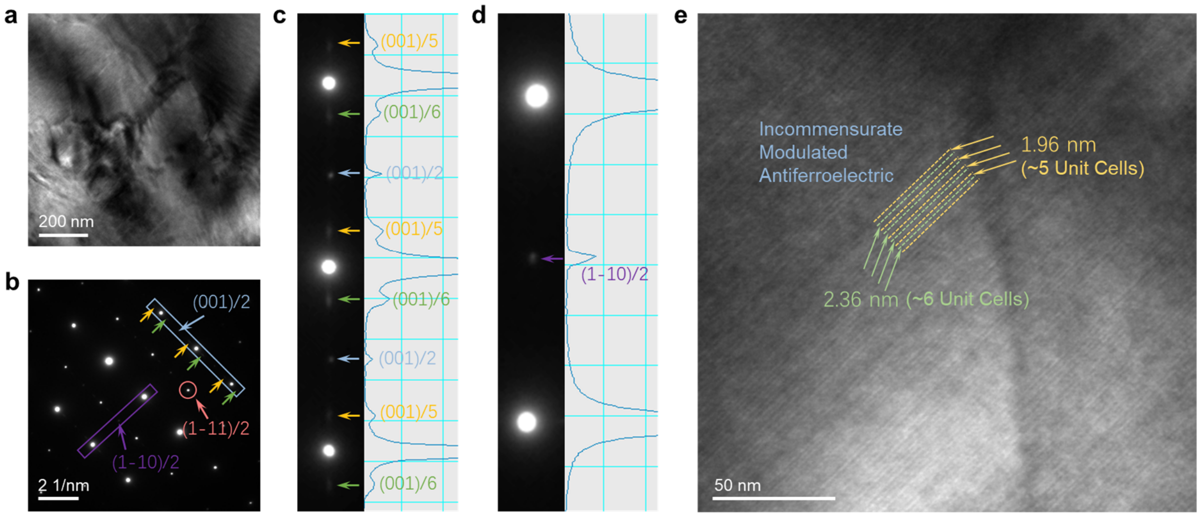

Supplementary Fig. S5 | Incommensurate modulated antiferroelectric structure for *x* = 0. **a**,**b**, (**a**) Domain morphology and (**b**) the corresponding SAED pattern. Yellow, green, blue, purple arrows and red circles represent the (001)/5, (001)/6, (001)/2, (1-10)/2, and (1-11)/2 superlattice diffraction points, respectively. **c**,**d**, Enlarged SAED patterns and the corresponding diffraction intensity of the marked (**c**) blue and (**d**) purple rectangles from (**b**). **e**, High-resolution TEM pattern of the amplified domain structure.

Striped nanodomains with weak contrast can be observed in $x = 0$ ceramic. The appearance of superlattices of (*ooe*)/2 and (*ooo*)/2 (*o* is odd and *e* is even) can demonstrate the coexisting in-phase and anti-phase BO_6 tilts according to the conclusions by *Glazer*[7]. We also find the (001)/5 and (001)/6 satellite spots from the SAED patterns, confirming the incommensurate modulation antiferroelectric structure in $x = 0$ ceramic. The incommensurate modulation is actually the average effect over an ensemble of stripes (or a mixture) of commensurate modulations[8], which is composed of stripes with periodicities of $n = 5$ and 6 in the $x = 0$ ceramic.

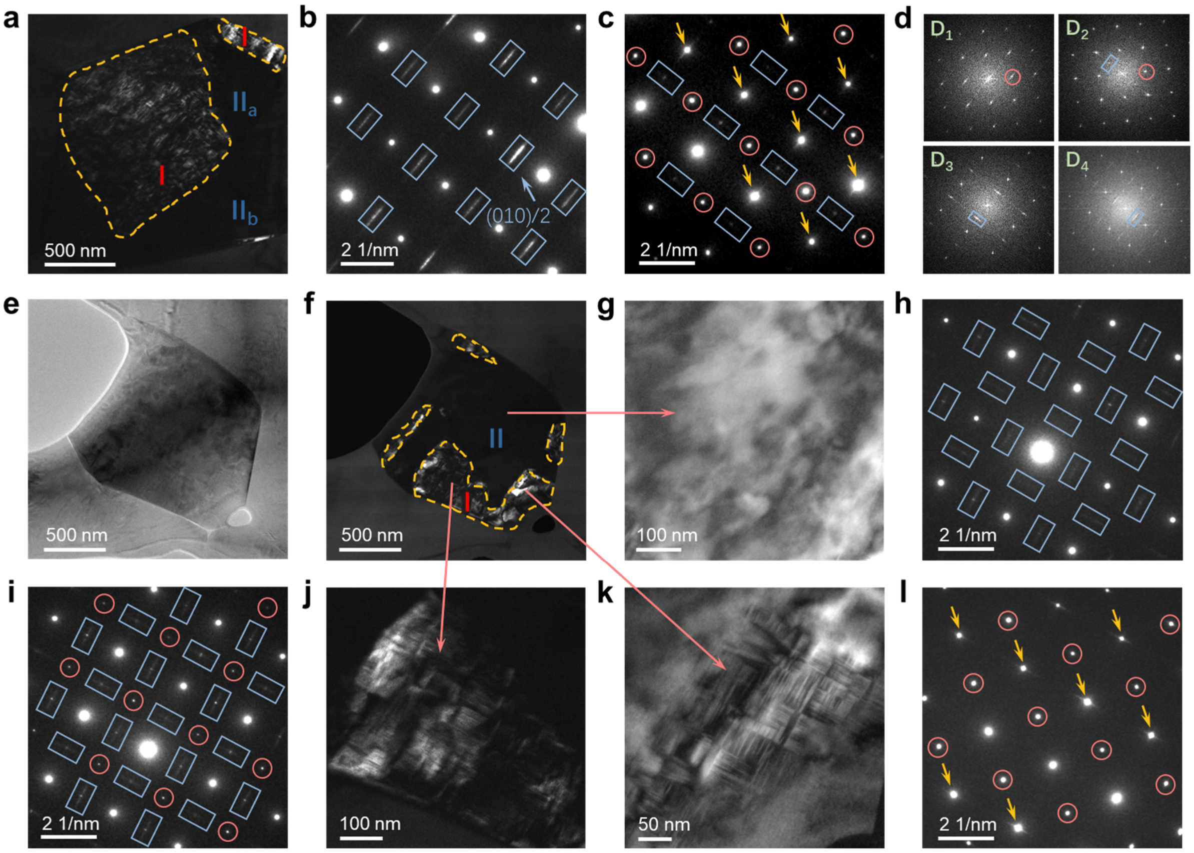

Supplementary Fig. S6 | Hierarchical heterostructure for $x = 0.25$. a, Heterostructure in one grain for $x = 0.25$ along $[100]_c$. **b**,**c**, SAED patterns of (**b**) region II_b and (**c**) the marked green square area from Fig. 2a. (**d**) Fast Fourier transform (FFT) patterns of the regions D_1, D_2, D_3, and D_4 from Fig. 2d. **e**,**f**, Heterostructure in another grain for $x = 0.25$. **g**,**h**, (**g**) Enlarged domain morphology and (**h**) the corresponding SAED pattern in region II from (**f**). **i**, SAED pattern from (**f**). **j**-**l**, (**j**,**k**) Amplified domain morphology and (**l**) the corresponding SAED pattern in region I from (**f**).

Zone D_1, D_3, and D_4 show the same FFT patterns of regions I, II_a, and II_b, respectively. Zone D_2 is the junction of regions I and II_b. The vertically intersecting striped nanodomains (region I) and disordered blotched domains (region II) can also be clearly and easily found in other grains, demonstrating the widespread existence of this heterostructure in $x = 0.25$ ceramic.

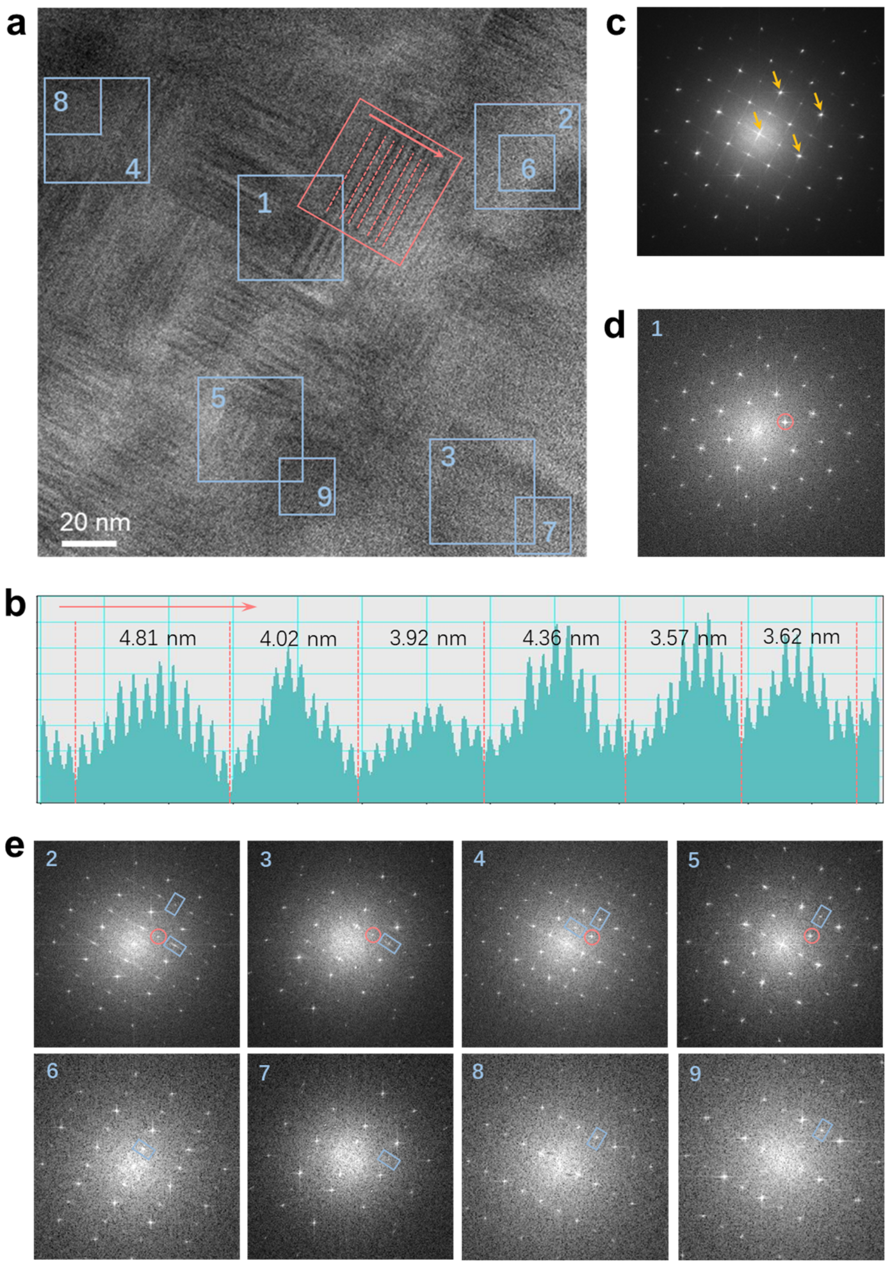

Supplementary Fig. S7 | Heterogeneous domain structure for *x* = 0.25. a, High-resolution TEM pattern of the amplified domain structure in region I from Supplementary Fig. S6f. **b**, The intensity and widths of stripes for the marked red rectangle from (**a**). **c**, SAED pattern of (**a**). **d**, FFT pattern of Zone 1 from (**a**). **e**, FFT pattern of Zone 2-9 from (**a**).

It can be clearly found that blotched nanodomains can be captured in the matrix of vertically intersecting striped nanodomains (region I), which means that the hetero nanodomain structure has been established in $x = 0.25$ ceramic. The stripe widths of the marked red rectangle indicate that the stripes are composed of 9-12 unit cells. In addition, we can also discover many finer or wider striped nanodomains.

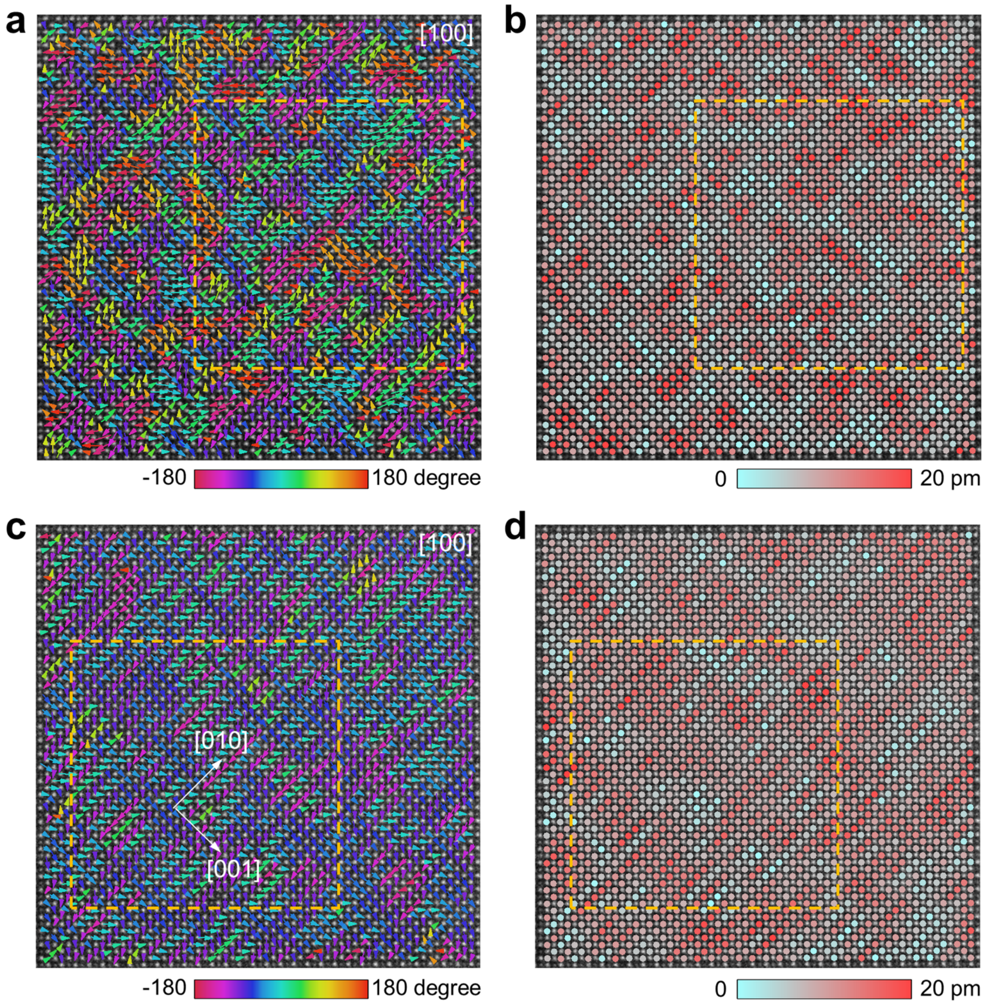

Supplementary Fig. S8 | Local polarization heterostructure for *x* = 0.25. a,b, Atomic-resolution HAADF-STEM image of the *x* = 0.25 ceramic with the corresponding (**a**) cation displacement vectors showing polarization interlocking structure and (**b**) polarization magnitudes for region I. **c,d**, Atomic-resolution HAADF-STEM image of the *x* = 0.25 ceramic with the corresponding (**c**) cation displacement vectors showing fishbone polarization configuration and (**d**) polarization magnitudes for region II. The yellow square areas of (**a**-**d**) are enlarged and displayed in Fig. 3a-d, respectively. Two-dimensional contours of polarization angles for regions I (**a**) and II (**c**) are shown in Fig. 3e,f.

It is noting that the two configurations of "polarization interlocking structure" and "fishbone polarization configuration" are defined based on the distribution characteristics and morphological features of their polarization vectors. In region I, these polarization nanoclusters are vertically interlaced and intertwined with each other, exhibiting a structural feature analogous to mutual interlocking. Thus, we term this configuration a "polarization interlocking structure". In region II, the alternating striped polarization regions with periodicities of n = 2 along $[001]_c$ and short-range alternating striped polarization regions with multiple periodicities along $[010]_c$ can be verified. These slender striped polarization regions are widely distributed, exhibiting a morphological feature analogous to fishbones, which can be well defined as "fishbone polarization structure".

These configurations cannot be categorized as PNRs or polymorphic nanodomains of relaxor ferroelectrics; instead, we term them "antiferroelectric nanoclusters". In relaxor ferroelectrics,

the polarization configuration typically exhibits as multiple randomly oriented PNRs embedded within non-polar matrices, where the polarization orientations of adjacent nanoregions tend to align along similar directions[9,10]. By contrast, in relaxor antiferroelectrics, antiferroelectric nanoclusters display nearly antiparallel polarization orientations in adjacent nanoregions, exhibiting characteristics of antiferroelectric polarization configurations. It is worth noting that due to the introduction of strong random fields formed by multiple components, the polarization vector in relaxor antiferroelectrics is not absolutely anti-parallel. Instead, both the magnitude and direction of polarization fluctuate within a finite range. Moreover, the commensurate modulated structure may also experience disturbances.

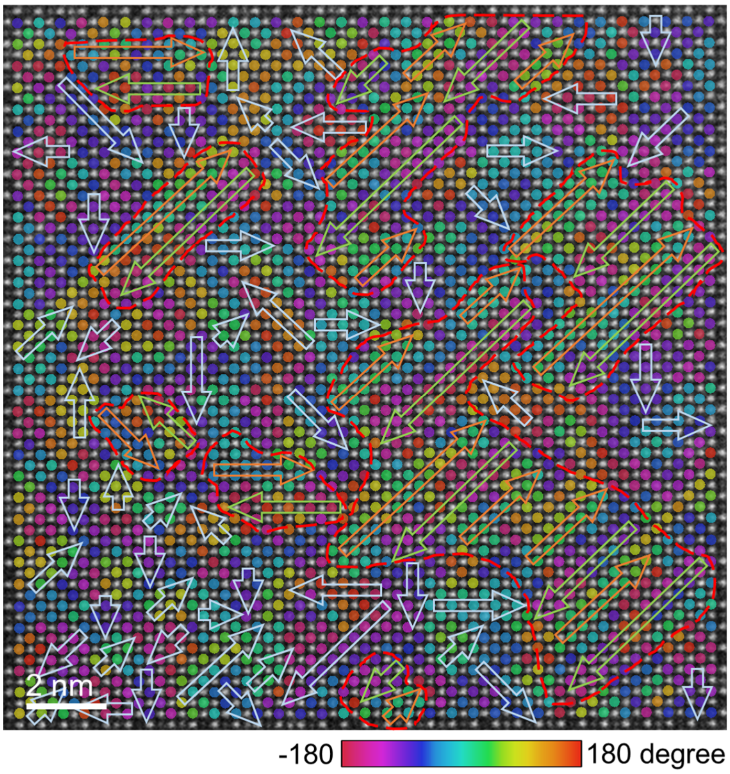

Supplementary Fig. S9 | Local polarization heterostructure for x = 0.25. The direction of hollow arrow represents the whole polarization direction of this region. Red dashed areas indicate the disturbed antiferroelectric regions exhibiting antiferroelectric nanoclusters.

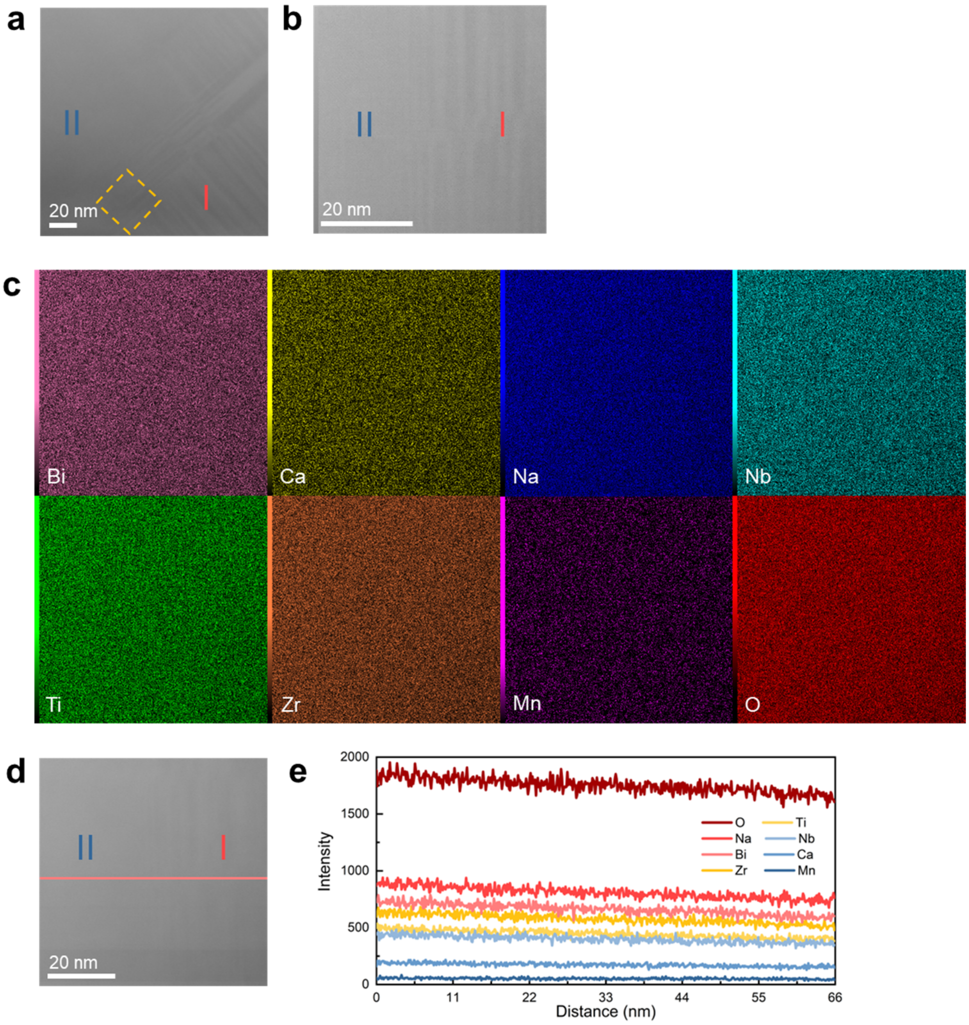

Supplementary Fig. S10 | Element distribution for x = 0.25. a, High-resolution TEM pattern of the boundary between regions I and II. **b**, High-resolution TEM pattern of the marked yellow square area from (**a**). **c**, Surface scanning of element distribution of (**b**). **d**,**e**, Line scanning map of element distribution (Red line form (**d**)).

According to the TEM scanning results of the elements, there is no obvious element segregation at the boundary of regions I and II in the x = 0.25 ceramic, showing a relatively uniform distribution behavior.

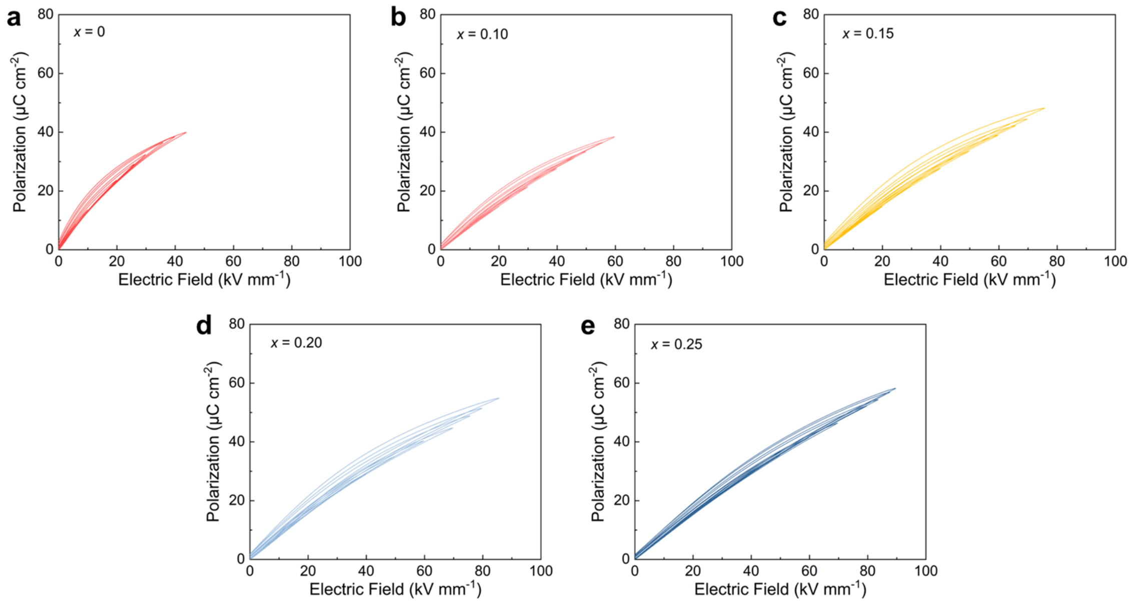

Supplementary Fig. S11 | *P*-*E* loops of NN-CZ-*x*BNT ceramics. a-e, *P*-*E* loops under different electric fields of (**a**) $x = 0$, (**b**) $x = 0.10$, (**c**) $x = 0.15$, (**d**) $x = 0.20$, and (**e**) $x = 0.25$.

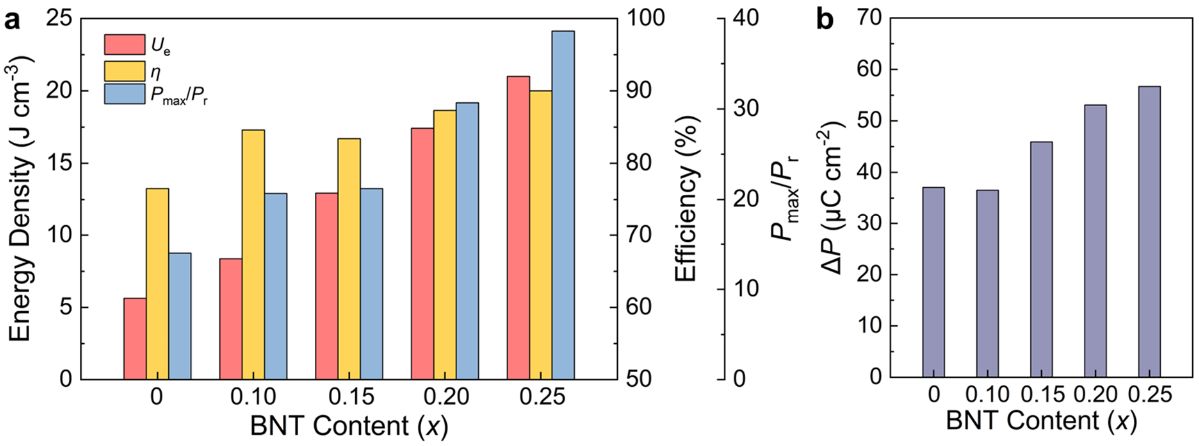

Supplementary Fig. S12 | Energy storage properties of NN-CZ-*x*BNT ceramics at breakdown electric fields. a,b, (**a**) Energy density, efficiency, P_{max}/P_r, and (**b**) ΔP of NN-CZ-*x*BNT ceramics. We can find that the U_e and η increase with increasing BNT.

The U_e and η for $x = 0$, $x = 0.10$, $x = 0.15$, $x = 0.20$, and $x = 0.25$ ceramics are 5.6 J cm^{-3} and 77%, 8.4 J cm^{-3} and 85%, 12.9 J cm^{-3} and 83%, 17.4 J cm^{-3} and 87%, 21.0 J cm^{-3} and 90%, respectively. The P_{max}/P_r for $x = 0$, $x = 0.10$, $x = 0.15$, $x = 0.20$, and $x = 0.25$ ceramics are 14.0, 20.7, 21.2, 30.7, and 38.6, respectively. The ΔP (P_{max} - P_r) for $x = 0$, $x = 0.10$, $x = 0.15$, $x = 0.20$, and $x = 0.25$ ceramics are 37.1, 36.5, 45.9, 53.1, and 56.7 μC cm^{-2}, respectively. The increased P_{max}/P_r and ΔP with increasing BNT means the improved polarization fluctuation behavior despite the significant increase in breakdown electric field, which contributes to the enhanced overall energy storage performance.

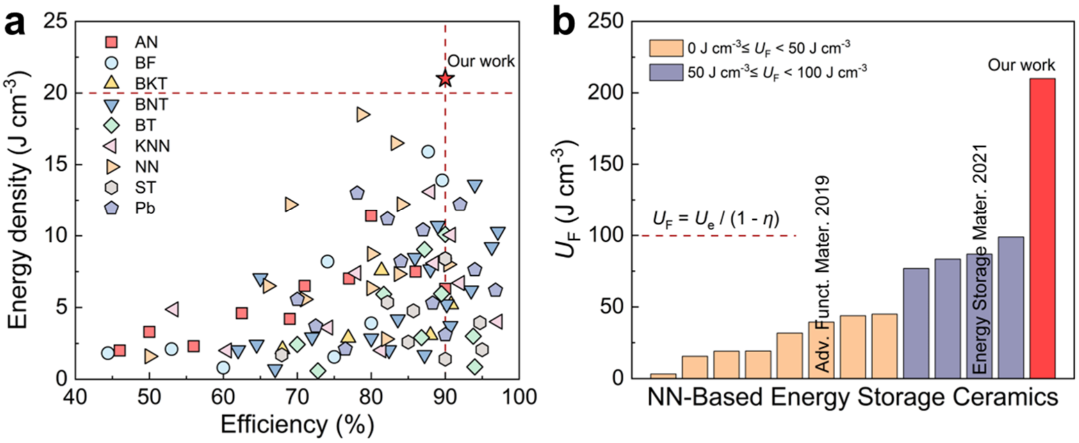

Supplementary Fig. S13 | Comparisons of comprehensive energy storage properties. a, Comparisons of energy density and efficiency between our ceramics and other representative lead-free and lead based systems. b, Comparisons of U_F between our ceramics and other representive NN-based energy storage ceramics.

It can be found that the U_e of most ceramics is no more than 10 J cm^{-3} when the η is maintained above 90%. It is known that the previous benchmark for the energy storage properties (U_e ~12.2 J cm^{-3} and η ~69%) of $NaNbO_3$-based antiferroelectric ceramics has been reported in 2019, exhibiting large progress in lead-free bulk ceramics[11]. Subsequently, in 2021, Jiang et al. reported ultrahigh energy storage density and enhanced efficiency (U_e ~18.5 J cm^{-3} and η ~78.7%) in $NaNbO_3$-based antiferroelectric ceramics[12], making the highest energy storage density among lead-free energy storage ceramics at that time. Although outstanding U_e have been reported, they all face the dilemma of low efficiency (η <80%), which can lead to serious energy dissipation. The challenge of achieving both ultrahigh U_e and ultrahigh η simultaneously has not been overcome in the following years. In this work, the U_e of 21.0 J cm^{-3} is realized in $x = 0.25$ lead-free antiferroelectric ceramic, which shows a huge breakthrough ($U_e \geq 20$ J cm^{-3}) in performance compared to the above reported $NaNbO_3$-based energy storage bulk ceramics, especially with ultrahigh efficiency ($\eta \geq 90\%$). To quantitatively evaluate the trade-off between U_e and η, the figure of merit $U_F = U_e / (1 - \eta)$ is applied to express the comprehensive energy storage properties[13,14]. Clearly, the $x = 0.25$ ceramic exhibits the highest U_F value of 210 J cm^{-3} among NN-based energy storage ceramics, indicating the optimal overall energy storage performance.

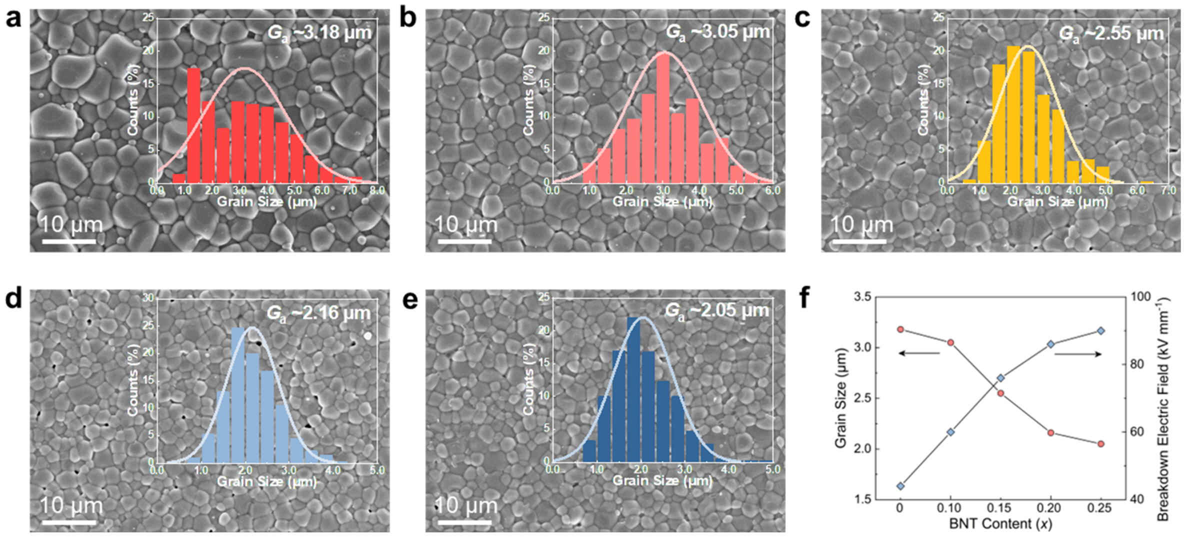

Supplementary Fig. S14 | SEM patterns and grain size distribution of NN-CZ-*x*BNT ceramics. a-e, SEM patterns and grain size distribution of (**a**) $x = 0$, (**b**) $x = 0.10$, (**c**) $x = 0.15$, (**d**) $x = 0.20$, and (**e**) $x = 0.25$. **f**, The change of average grain size and breakdown electric field with increasing BNT.

All ceramics show dense microstructures without large pores. The average grain size G_a of $x = 0$, $x = 0.10$, $x = 0.15$, $x = 0.20$, and $x = 0.25$ ceramics are 3.18, 3.05, 2.55, 2.16, and 2.05 μm, respectively. The decreased G_a contributes to the enhanced E_b (44, 60, 76, 86, and 90 kV mm^{-1} for $x = 0$, $x = 0.10$, $x = 0.15$, $x = 0.20$, and $x = 0.25$ ceramics) based on an exponential decay relationship of $E_b \propto 1/\sqrt{G_a}$[15].

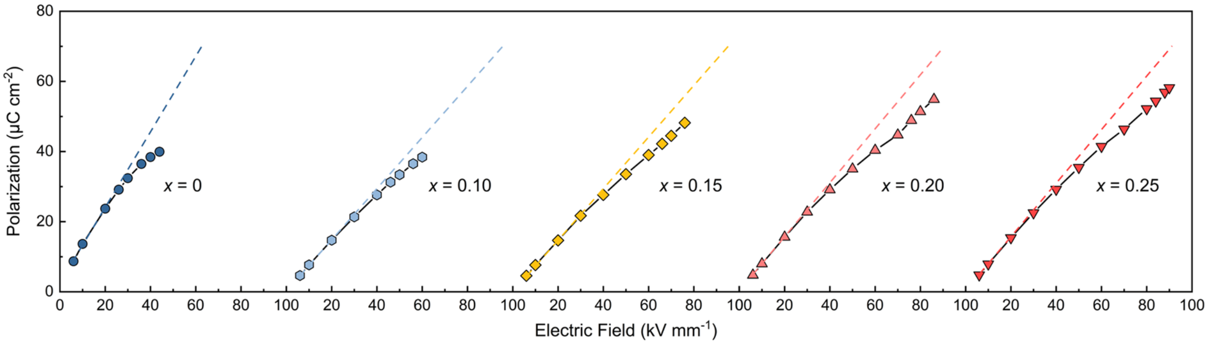

Supplementary Fig. S15 | Maximum polarization of NN-CZ-*x*BNT ceramics as a function of electric field.

The phenomenon of polarization saturation can be obviously found in $x = 0$ ceramic accompanied by the premature deviation of polarization from low electric field slope. As BNT increases, the polarization saturation phenomenon is suppressed. Surprisingly, polarization exhibits an abnormal behavior of significant improvement with increasing electric field under high electric fields. The behavior can be clearly shown in the non-overlapping charging process under different electric fields from *P*-*E* loops of $x = 0.10$, $x = 0.15$, $x = 0.20$, and $x = 0.25$ ceramics (Supplementary Fig. S11), which overcomes the common behavior of polarization increasing slowly with the increase of electric fields, significantly enhancing P_{max}.

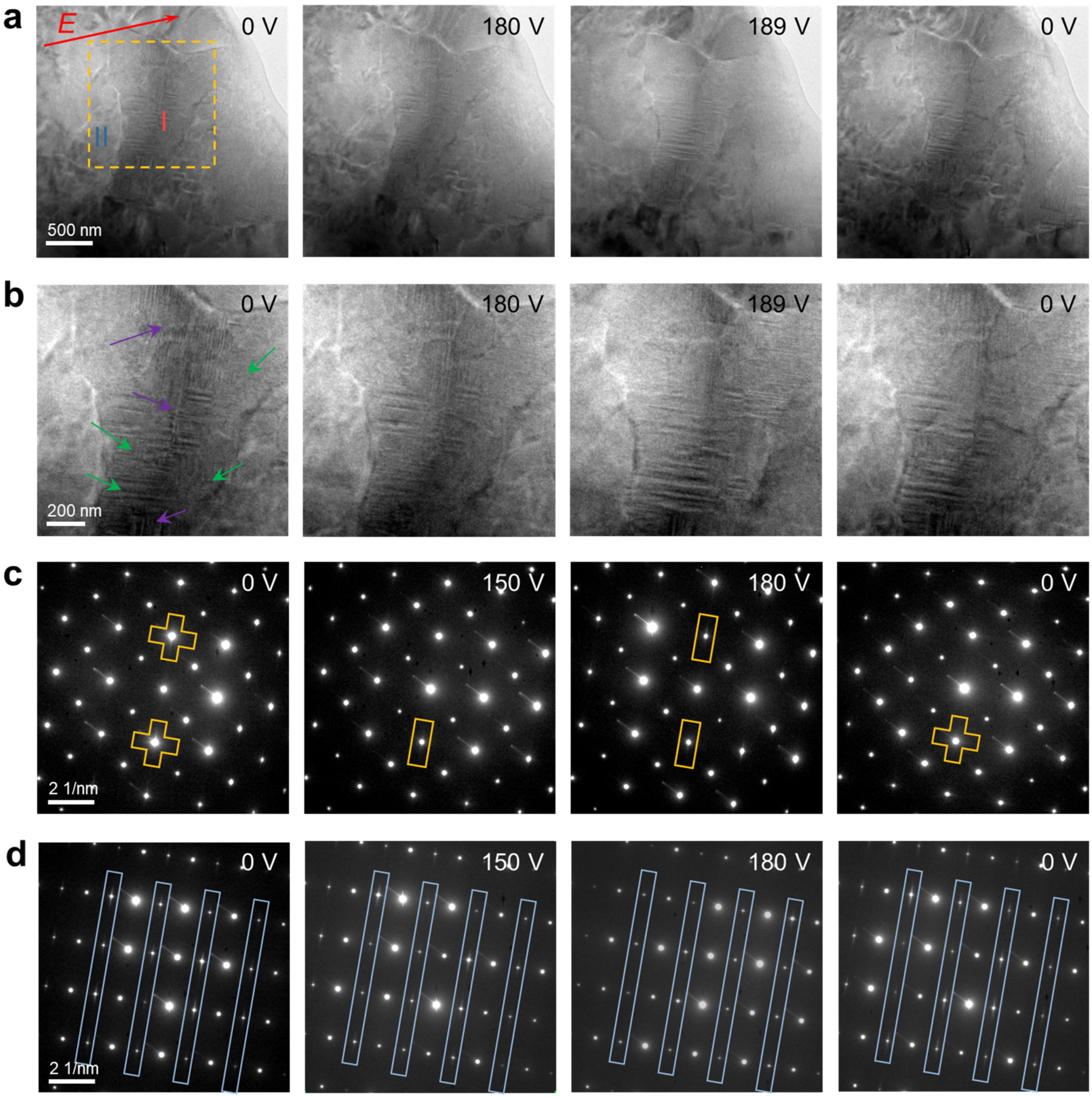

Supplementary Fig. S16 | In situ TEM images in heterostructure region of $x = 0.25$ ceramic during electric field loading and unloading processes. **a,** The evolution of domain structures from 0 → 180 → 189 → 0 V. The red arrow indicates the direction in which the external electric field is applied. **b,** The evolution of domain structures of the marked yellow square area from (**a**). The green and purple arrows point to the evolution zones of horizontal and vertical striped domains, respectively. **c**,**d** The evolution of SAED patterns in regions (**c**) I and (**d**) II from 0 → 150 → 180 → 0 V. The yellow hollow crosses represent the antiferroelectric satellite points along the $[010]_c$ and $[001]_c$ directions. Yellow rectangles represent the antiferroelectric satellite points along the $[010]_c$ direction. Blue rectangles represent (010)/2 superlattice diffraction points.

It can be clearly found that the striped nanodomains close to the direction of the electric field grow with increasing electric field, while the striped domains perpendicular to the direction of the electric field decrease and even disappear with increasing electric field, which are related to the gradual release of polarization interlock driven by electric field. The transformation of antiferroelectric satellite points along the $[010]_c$ and $[001]_c$ directions into satellite points along one direction as the electric field increases also indicates the weakening of the interlocked

polarization structure under the action of the electric field in region I. It should be noted that polarization interlocking structure cannot be effectively released under low electric fields, manifested as vertical striped nanodomains and antiferroelectric satellite points that still exist under low electric fields, which requires ultrahigh electric fields to be fully released. This behavior contributes to the anomalously increasing polarization and delayed polarization saturation with increasing electric field, especially under high electric fields. Furthermore, the satellite points perpendicular to the (010)/2 superlattice points gradually weaken and disappear as the electric field increases, indicating a transition from fishbone polarization configuration to ferroelectric polarization order, which also contributes to the enhancement of P_{m}. When the electric field is removed, the domain morphologies and SAED patterns can be effectively restored to the initial state, demonstrating the high recoverability of antiferroelectric nanoclusters with polarization interlocking and fishbone polarization structures, which can effectively optimize polarization recovery path, reducing P_{r} and improving η.

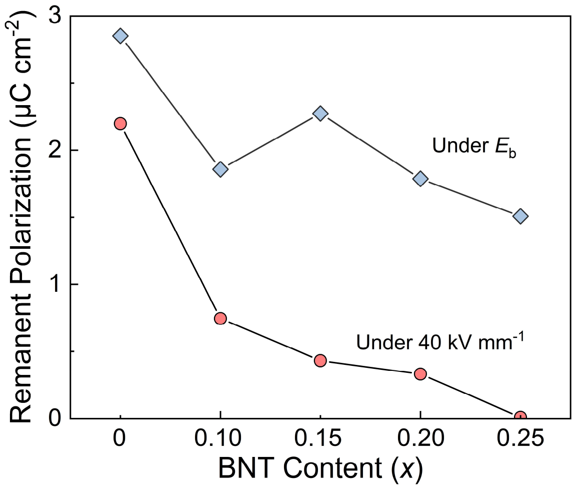

Supplementary Fig. S17 | Remanent polarization of NN-CZ-*x*BNT ceramics under electric field of 40 kV mm^{-1} and E_b.

The largely decreased P_r with increasing BNT can be clearly discovered under the same electric field of 40 kV mm^{-1}. The negligible P_r is mainly attributed to the polarization interlocking structure, which is formed by the embedding polarization regions with different orientations on the basis of anti-parallel polarizations or incommensurate modulation antiferroelectric regions. Polarization interlocking structure can provide polarization restoring force after removing the electric field (discharging progress), promoting polarization to quickly and efficiently return to the initial state, which can significantly reduce P_r and improve η. It is worth mentioning that the hierarchical heterostructures with interlocked polarization feature effectively eliminates the polarization hysteresis caused by the (diffuse) antiferroelectric-ferroelectric phase transition in (relaxor) antiferroelectrics. Ultralow P_r can also be found in x = 0.25 ceramic under ultrahigh E_b of 90 kV mm^{-1}, which is much higher than that (44 kV mm^{-1}) of x = 0 ceramic.

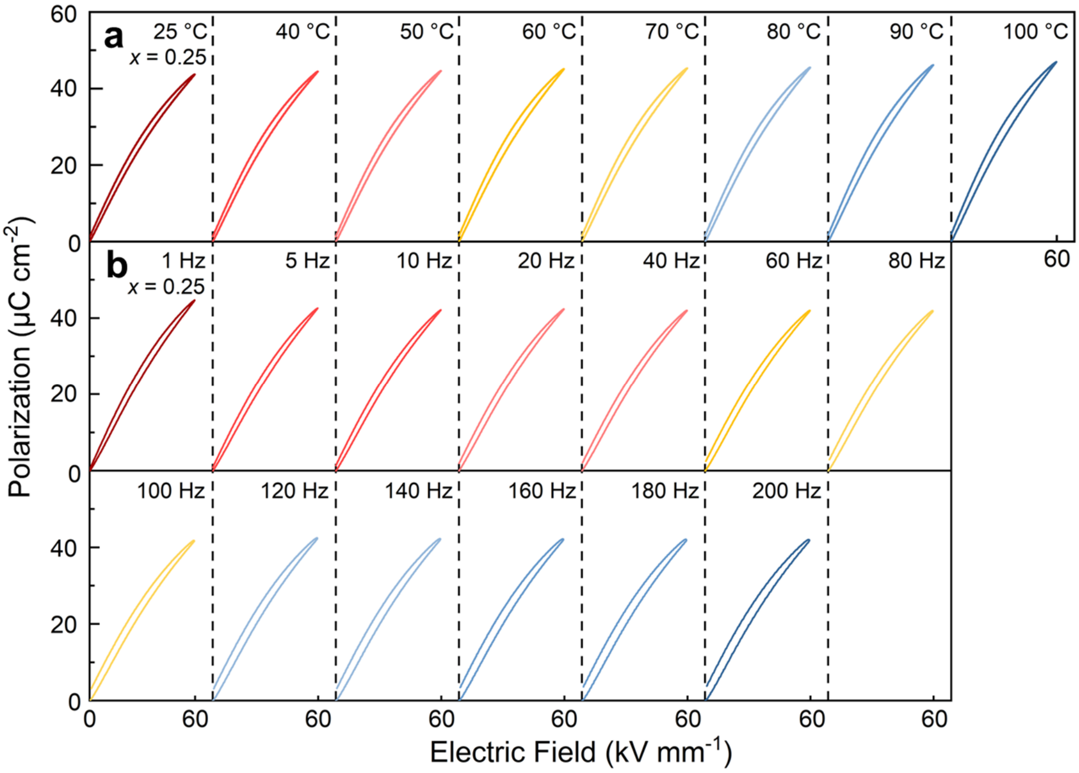

Supplementary Fig. S18 | Temperature and frequency stability of *x* = 0.25. a, Temperature-dependent *P*-*E* loops at 10 Hz under an electric field of 60 kV mm^{-1}. **b**, Frequency-dependent room-temperature *P*-*E* loops under an electric field of 60 kV mm^{-1}.

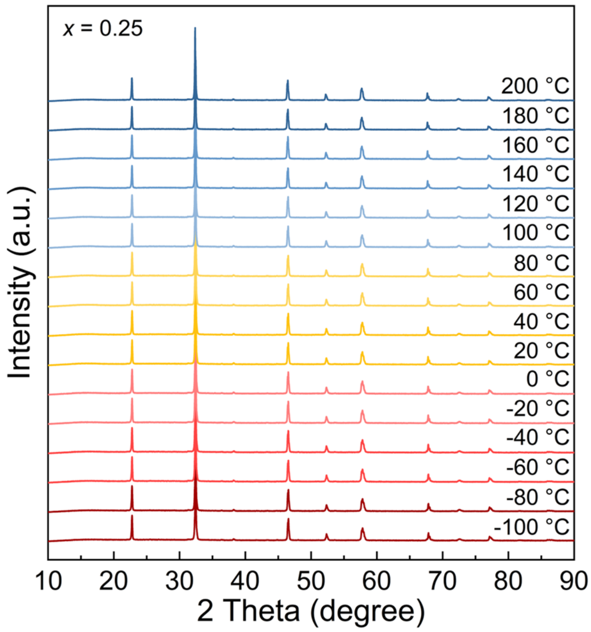

Supplementary Fig. S19 | Temperature-dependent X-ray diffraction patterns for *x* = 0.25.

The *x* = 0.25 ceramic shows the temperature insensitive phase structure without the changes in the number and position of diffraction peaks.

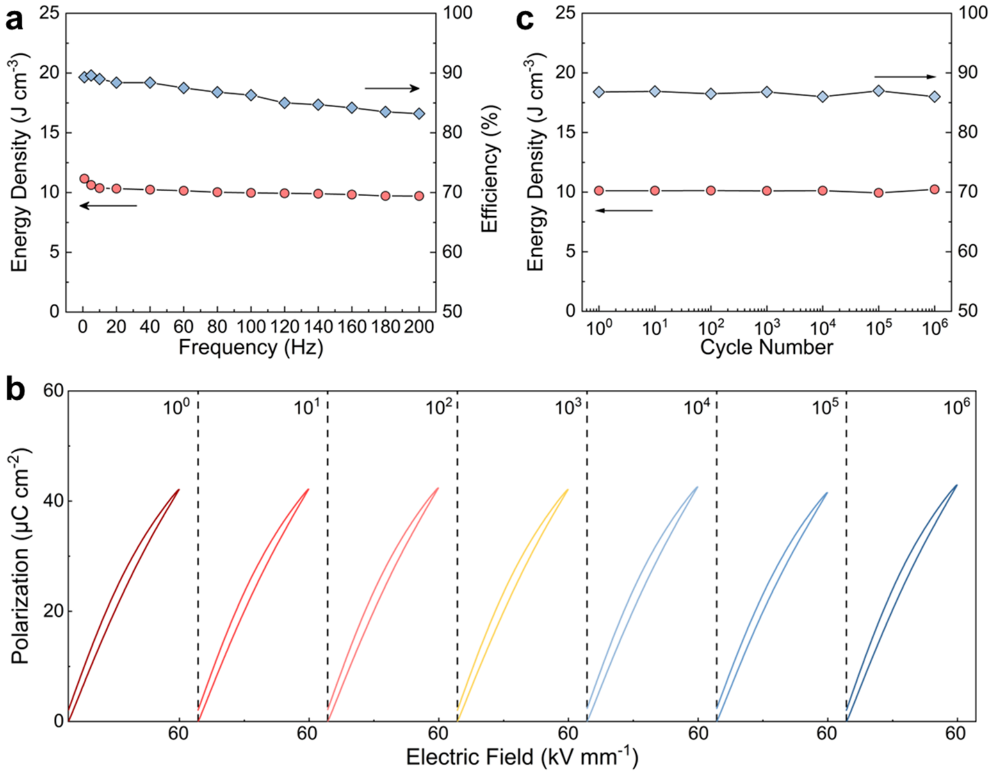

Supplementary Fig. S20 | Frequency and cycling stability of *x* = 0.25. a, Frequency-dependent energy storage performance at an electric field of 60 kV mm^{-1}. **b**,**c**, (**b**) *P*-*E* loops and (**c**) U_e and η as a function of cycle number at an electric field of 60 kV mm^{-1}.

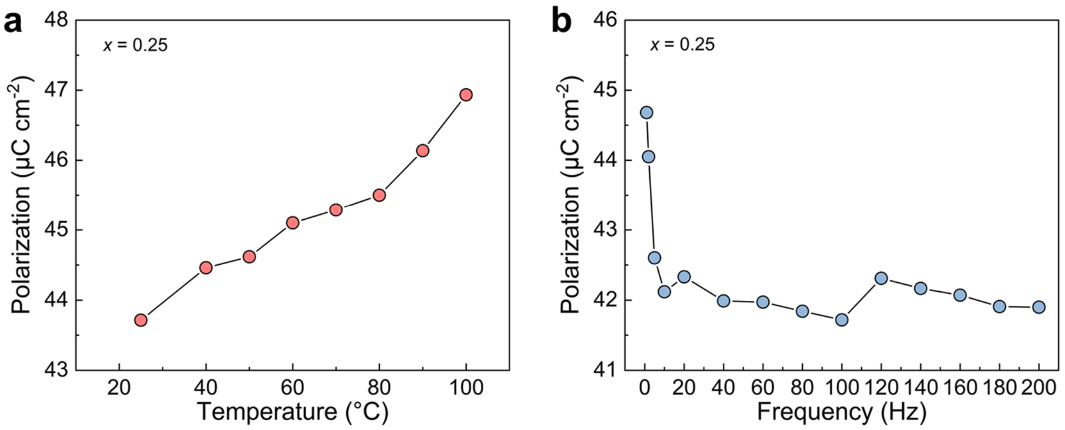

Supplementary Fig. S21 | Maximum polarization as a function of temperature and frequency for x = 0.25. a, Polarization as a function of temperature at an electric field of 60 kV mm^{-1}. **b**, Polarization as a function of frequency at an electric field of 60 kV mm^{-1}.

The increased P_m can compensate for the slight decrease in η, maintaining stable U_e with increasing temperature.

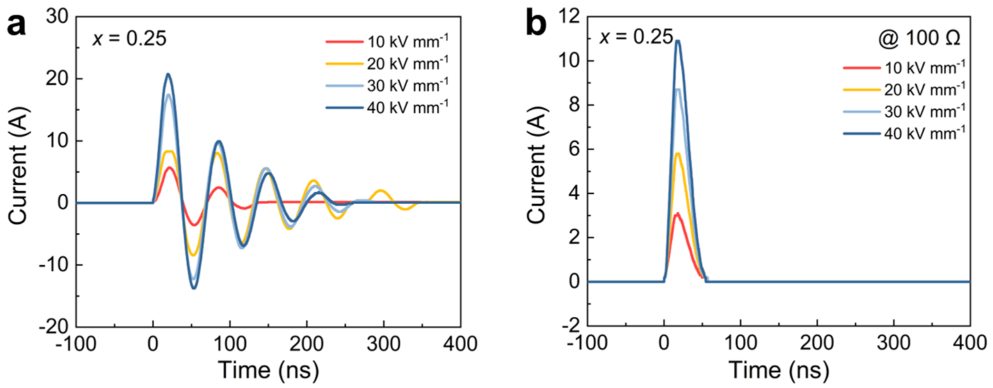

Supplementary Fig. S22 | Charge-discharge performance for *x* = 0.25. a, Underdamped discharge waveforms under different electric fields. **b**, Overdamped discharge waveforms under different electric fields with a fixed resistor (R = 100 Ω).

The power density P_D and discharge energy density U_D can be calculated using the following formulas:

$$P_D = \frac{E \times I_{max}}{2S} \qquad (S1)$$

$$U_D = \frac{R \int I_{(t)}^2 dt}{V} \qquad (S2)$$

where S and V is the electrode area and sample volume, respectively.

Supplementary Table S1. Relevant references for Fig. 4b.

Data	References
Figure 4B	[9,10,14,16-98]

Supplementary Table S2. A comparison of the comprehensive performance between NN-CZ-0.25BNT ceramic and other representive lead-free energy storage ceramics.

Compositions	U_e (J cm^{-3})	η (%)	E_b (kV mm^{-1})	$t_{0.9}$ (ns)	Ref.
0.88(0.94$NaNbO_3$-0.06$BiFeO_3$)-0.12$Sr_{0.7}Bi_{0.2}\square_{0.1}Ti_{0.75}Ta_{0.2}\square_{0.05}O_3$	12.25	83	78	88	[99]
0.85$BaTiO_3$-0.15$(Bi_{0.5}Na_{0.5})(Zn_{1/3}Nb_{2/3})O_3$	11.6	96.1	58	41	[14]
0.848$(Na_{0.52}K_{0.48})(Sb_{0.035}Nb_{0.965})O_3-0.012SrZrO_3-0.14(Bi_{0.5}Na_{0.5})ZrO_3$	13.1	90	74	35.2	[100]
0.62$Bi_{0.9}La_{0.1}FeO_3$-0.3$Ba_{0.7}Sr_{0.3}TiO_3$-0.08$NaNb_{0.85}Ta_{0.15}O_3$	15.9	87.7	68	5700	[101]
0.7$Bi_{0.47}Na_{0.47}Ba_{0.06}TiO_3-0.3Sr_{0.7}La_{0.2}Ta_{0.2}Ti_{0.75}O_3$	15.48	90.02	71	33	[102]
$Bi_{0.25}Na_{0.25}Ba_{0.5}Ti_{0.92}Hf_{0.08}O_3$	16.21	90.5	80	30.6	[103]
$Bi_{0.2}Na_{0.2}K_{0.2}La_{0.2}Sr_{0.2}(Ti_{0.95}Nb_{0.05})O_3$	16.4	90	85	20	[104]
$(Bi_{0.35}K_{0.175}Ba_{0.3}Na_{0.175})(Ti_{0.7}Zr_{0.3})O_3$	17.3	88.5	78	30	[105]
0.90$NaNbO_3$-0.10$BiFeO_3$	18.5	78.7	91	14	[12]
NN-CZ-0.25BNT	21.0	90	90	32	Our work

References

1 Qi, H. *et al.* Large (anti)ferrodistortive $NaNbO_3$-based lead-free relaxors: polar nanoregions embedded in ordered oxygen octahedral tilt matrix. *Mater. Today* **60**, 91-97 (2022).

2 Chen, L. *et al.* Local diverse polarization optimized comprehensive energy-storage performance in lead-free superparaelectrics. *Adv. Mater.* **34**, 2205787 (2022).

3 Yang, Z. *et al.* Realizing high comprehensive energy storage performance in lead-free bulk ceramics via designing an unmatched temperature range. *J. Mater. Chem. A* **7**, 27256-27266 (2019).

4 Pan, H. *et al.* Ultrahigh energy storage in superparaelectric relaxor ferroelectrics. *Science* **374**, 100-104 (2021).

5 Zhang, M., Yang, H., Lin, Y., Yuan, Q. & Du, H. Significant increase in comprehensive energy storage performance of potassium sodium niobate-based ceramics via synergistic optimization strategy. *Energy Storage Mater.* **50**, 563-563 (2022).

6 Long, C. *et al.* Simultaneously realizing ultrahigh energy storage density and efficiency in $BaTiO_3$-based dielectric ceramics by creating highly dynamic polar nanoregions and intrinsic conduction. *Acta Mater.* **256**, 119135 (2023).

7 Glazer, A. Simple ways of determining perovskite structures. *Acta Cryst. A* **31**, 756-762 (1975).

8 Tan, X., Ma, C., Frederick, J., Beckman, S. & Webber, K. G. The antiferroelectric ↔ ferroelectric phase transition in lead-containing and lead-free perovskite ceramics. *J. Am. Ceram. Soc.* **94**, 4091-4107 (2011).

9 Chen, L. *et al.* Giant energy-storage density with ultrahigh efficiency in lead-free relaxors via high-entropy design. *Nat. Commun.* **13**, 3089 (2022).

10 Li, D. *et al.* A high-temperature performing and near-zero energy loss lead-free ceramic capacitor. *Energy Environ. Sci.* **16**, 4511-4521 (2023).

11 Qi, H. *et al.* Ultrahigh energy-storage density in $NaNbO_3$-based lead-free relaxor antiferroelectric ceramics with nanoscale domains. *Adv. Funct. Mater.* **29**, 1903877 (2019).

12 Jiang, J. *et al.* Ultrahigh energy storage density in lead-free relaxor antiferroelectric ceramics via domain engineering. *Energy Storage Mater.* **43**, 383-390 (2021).

13 Belhadi, J. *et al.* Ultra-high energy storage density and efficiency at low electric fields/voltages in dielectric thin film capacitors through synergistic effects. *J. Materiomics* **11**, 100980 (2025).

14 Chen, L. *et al.* Near-zero energy consumption capacitors by controlling inhomogeneous polarization configuration. *Adv. Mater.* **36**, 2313285 (2024).

15 Tunkasiri, T. & Rujijanagul, G. Dielectric strength of fine grained barium titanate ceramics. *J. Mater. Sci. Lett.* **15**, 1767-1769 (1996).

16 Li, L. *et al.* Preparation and dielectric properties of $BaCu(B_2O_5)$-doped $SrTiO_3$-based ceramics for energy storage. *Mater. Sci. Eng. B* **178**, 1509-1514 (2013).

17 Wang, T., Jin, L., Li, C., Hu, Q. & Wei, X. Relaxor ferroelectric $BaTiO_3$-$Bi(Mg_{2/3}Nb_{1/3})O_3$ ceramics for energy storage application. *J. Am. Ceram. Soc.* **98**, 559-566 (2015).

18 Sun, Z., Li, L., Yu, S., Kang, X. & Chen, S. Energy storage properties and relaxor

behavior of lead-free $Ba_{1-x}Sm_{2x/3}Zr_{0.15}Ti_{0.85}O_3$ ceramics. *Dalton Trans.* **46**, 14341-14347 (2017).
19 Chen, J., Si, F., Zhao, P., Zhang, S. & Tang, B. Novel lead-free (1-*x*)$Sr_{0.7}Bi_{0.2}TiO_3$-*x*$La(Mg_{0.5}Zr_{0.5})O_3$ energy storage ceramics with high charge-discharge and excellent temperature-stable dielectric properties. *Ceram. Int.* **47**, 26215-26223 (2021).
20 Jiang, X. *et al.* Enhanced energy storage and fast discharge properties of $BaTiO_3$ based ceramics modified by $Bi(Mg_{1/2}Zr_{1/2})O_3$. *J. Eur. Ceram. Soc.* **39**, 1103-1109 (2019).
21 Yang, H. *et al.* A lead free relaxation and high energy storage efficiency ceramics for energy storage applications. *J. Alloys Compd.* **710**, 436-445 (2017).
22 Wang, J., Fan, H., Hu, B. & Jiang, H. Enhanced energy-storage performance and temperature-stable dielectric properties of (1-*x*)($0.94Na_{0.5}Bi_{0.5}TiO_3$-$0.06BaTiO_3$)-*x*$Na_{0.73}Bi_{0.09}NbO_3$ ceramics. *J. Mater. Sci.: Mater. Electron.* **30**, 2479-2488 (2019).
23 Zhu, C., Cai, Z., Li, L. & Wang, X. High energy density, high efficiency and excellent temperature stability of lead free Mn-doped $BaTiO_3$-$Bi(Mg_{1/2}Zr_{1/2})O_3$ ceramics sintered in a reducing atmosphere. *J. Alloys Compd.* **816**, 152498 (2020).
24 Zhang, L., Pang, L.-X., Li, W.-B. & Zhou, D. Extreme high energy storage efficiency in perovskite structured (1-*x*)$(Ba_{0.8}Sr_{0.2})TiO_3$-*x*$Bi(Zn_{2/3}Nb_{1/3})O_3$ ($0.04 \leqslant x \leqslant 0.16$) ceramics. *J. Eur. Ceram. Soc.* **40**, 3343-3347 (2020).
25 Wang, L.-M., Liu, Q.-X. & Zhou, D. Dielectric and energy storage properties of the (1-*x*)$BaTiO_3$-*x*$Bi(Li_{1/3}Hf_{2/3})O_3$ ($0.08 \leq x \leq 0.14$) ceramics. *Mater. Lett.* **283**, 128823 (2021).
26 Han, D. *et al.* Ultrahigh energy efficiency of (1-*x*)$Ba_{0.85}Ca_{0.15}Zr_{0.1}Ti_{0.9}O_3$-*x*$Bi(Mg_{0.5}Sn_{0.5})O_3$ lead-free ceramics. *J. Alloys Compd.* **902**, 163721 (2022).
27 Li, W.-B., Zhou, D. & Pang, L.-X. Enhanced energy storage density by inducing defect dipoles in lead free relaxor ferroelectric $BaTiO_3$-based ceramics. *Appl. Phys. Lett.* **110**, 132902 (2017).
28 Wang, X., Wu, X., Yang, D., Yin, J. & Wu, J. Achieving superior energy-storage efficiency by tailoring the state of polar nano-sized regions under low electric fields. *Chem. Eng. J.* **447**, 137494 (2022).
29 Yang, H., Yan, F., Lin, Y. & Wang, T. Enhanced energy storage properties of $Ba_{0.4}Sr_{0.6}TiO_3$ lead-free ceramics with Bi_2O_3-B_2O_3-SiO_2 glass addition. *J. Eur. Ceram. Soc.* **38**, 1367-1373 (2018).
30 Dai, Z. *et al.* Enhanced energy storage properties and stability of $Sr(Sc_{0.5}Nb_{0.5})O_3$ modified $0.65BaTiO_3$-$0.35Bi_{0.5}Na_{0.5}TiO_3$ ceramics. *Chem. Eng. J.* **397**, 125520 (2020).
31 Huang, Y., Zhao, C., Wu, B. & Wu, J. Multifunctional $BaTiO_3$-based relaxor ferroelectrics toward excellent energy storage performance and electrostrictive strain benefiting from crossover region. *ACS Appl. Mater. Interfaces* **12**, 23885-23895 (2020).
32 Wang, W. *et al.* Combining high energy efficiency and fast charge-discharge capability in calcium strontium titanate-based linear dielectric ceramic for energy-storage. *Ceram. Int.* **46**, 11484-11491 (2020).
33 Zhou, M., Liang, R., Zhou, Z. & Dong, X. Combining high energy efficiency and fast charge-discharge capability in novel $BaTiO_3$-based relaxor ferroelectric ceramic for energy-storage. *Ceram. Int.* **45**, 3582-3590 (2019).
34 Wang, H. *et al.* Enhanced energy density and discharged efficiency of lead-free relaxor (1-*x*)$[(Bi_{0.5}Na_{0.5})_{0.94}Ba_{0.06}]_{0.98}La_{0.02}TiO_3$-*x*$KNb_{0.6}Ta_{0.4}O_3$ ceramic capacitors. *Chem. Eng. J.* **394**, 124879 (2020).

35 Zhao, P. *et al.* Novel Ca doped $Sr_{0.7}Bi_{0.2}TiO_3$ lead-free relaxor ferroelectrics with high energy density and efficiency. *J. Eur. Ceram. Soc.* **40**, 1938-1946 (2020).

36 Zhou, M., Liang, R., Zhou, Z. & Dong, X. Novel $BaTiO_3$-based lead-free ceramic capacitors featuring high energy storage density, high power density, and excellent stability. *J. Mater. Chem. C* **6**, 8528-8537 (2018).

37 Si, F., Tang, B., Fang, Z., Li, H. & Zhang, S. A new type of $BaTiO_3$-based ceramics with $Bi(Mg_{1/2}Sn_{1/2})O_3$ modification showing improved energy storage properties and pulsed discharging performances. *J. Alloys Compd.* **819**, 153004 (2020).

38 Chen, Z. *et al.* Novel BCZT-based ceramics with ultrahigh energy storage efficiency and outstanding high temperature fatigue endurance and stability for practical application. *Ceram. Int.* **49**, 34520-34528 (2023).

39 Chen, Z. *et al.* Simultaneously achieving high energy storage density and efficiency under low electric field in $BiFeO_3$-based lead-free relaxor ferroelectric ceramics. *J. Eur. Ceram. Soc.* **40**, 5450-5457 (2020).

40 Wang, Q., Gong, P.-M. & Wang, C.-M. High recoverable energy storage density and large energy efficiency simultaneously achieved in $BaTiO_3$-$Bi(Zn_{1/2}Zr_{1/2})O_3$ relaxor ferroelectrics. *Ceram. Int.* **46**, 22452-22459 (2020).

41 Chen, X. *et al.* Achieving ultrahigh energy storage density and energy efficiency simultaneously in barium titanate based ceramics. *Appl. Phys. A* **126**, 146 (2020).

42 Li, J., Li, F., Xu, Z. & Zhang, S. Multilayer lead-free ceramic capacitors with ultrahigh energy density and efficiency. *Adv. Mater.* **30**, 1802155 (2018).

43 Si, F., Tang, B., Fang, Z., Li, H. & Zhang, S. Enhanced energy storage and fast charge-discharge properties of $(1-x)BaTiO_3$-$xBi(Ni_{1/2}Sn_{1/2})O_3$ relaxor ferroelectric ceramics. *Ceram. Int.* **45**, 17580-17590 (2019).

44 Zhang, Y. *et al.* Enhancement of energy storage performance in lead-free relaxor ferroelectric ceramics via band structure engineering. *J. Am. Ceram. Soc.* **106**, 6630-6640 (2023).

45 Kang, R. *et al.* Domain engineered lead-free ceramics with large energy storage density and ultra-high efficiency under low electric fields. *ACS Appl. Mater. Interfaces* **13**, 25143-25152 (2021).

46 Ning, Y. *et al.* Achieving high energy storage performance below 200 kV/cm in $BaTiO_3$-based medium-entropy ceramics. *Ceram. Int.* **49**, 20326-20333 (2023).

47 Li, X., Chen, X., Sun, J., Zhou, M. & Zhou, H. Novel lead-free ceramic capacitors with high energy density and fast discharge performance. *Ceram. Int.* **46**, 3426-3432 (2020).

48 Kong, X., Yang, L., Cheng, Z. & Zhang, S. Bi-modified $SrTiO_3$-based ceramics for high-temperature energy storage applications. *J. Am. Ceram. Soc.* **103**, 1722-1731 (2020).

49 Huang, Y. *et al.* $(Bi_{0.51}Na_{0.47})TiO_3$ based lead free ceramics with high energy density and efficiency. *J. Materiomics* **5**, 385-393 (2019).

50 Huang, Y., Guo, Q., Hao, H., Liu, H. & Zhang, S. Tailoring properties of $(Bi_{0.51}Na_{0.47})TiO_3$ based dielectrics for energy storage applications. *J. Eur. Ceram. Soc.* **39**, 4752-4760 (2019).

51 Wang, Y. *et al.* Structure, dielectric properties of novel $Ba(Zr,Ti)O_3$ based ceramics for energy storage application. *Ceram. Int.* **46**, 12080-12087 (2020).

52 Chen, X. *et al.* Simultaneously achieving ultrahigh energy storage density and energy efficiency in barium titanate based ceramics. *Ceram. Int.* **46**, 2764-2771 (2020).

53 Pu, Y. *et al.* Enhancing the energy storage properties of $Ca_{0.5}Sr_{0.5}TiO_3$-based lead-free linear dielectric ceramics with excellent stability through regulating grain boundary defects. *J. Mater. Chem. C* **7**, 14384-14393 (2019).
54 Yang, L., Kong, X., Cheng, Z. & Zhang, S. Ultra-high energy storage performance with mitigated polarization saturation in lead-free relaxors. *J. Mater. Chem. A* **7**, 8573-8580 (2019).
55 Zhou, M., Liang, R., Zhou, Z. & Dong, X. Achieving ultrahigh energy storage density and energy efficiency simultaneously in sodium niobate-based lead-free dielectric capacitors via microstructure modulation. *Inorg. Chem. Front.* **6**, 2148-2157 (2019).
56 Wang, J. *et al.* An alternative way to design excellent energy-storage properties in $Na_{0.5}Bi_{0.5}TiO_3$-based lead-free system by constructing relaxor dielectric composites. *J. Eur. Ceram. Soc.* **42**, 6512-6517 (2022).
57 Ding, Y. *et al.* Simultaneously achieving high energy-storage efficiency and density in Bi-modified $SrTiO_3$-based relaxor ferroelectrics by ion selective engineering. *Compos. Part B: Eng.* **230**, 109493 (2022).
58 Zhang, X. *et al.* Enhancement of recoverable energy density and efficiency of lead-free relaxor-ferroelectric BNT-based ceramics. *Chem. Eng. J.* **406**, 126818 (2021).
59 Liu, L. *et al.* Achieving high energy storage performance and ultrafast discharge speed in $SrTiO_3$-based ceramics via a synergistic effect of chemical modification and defect chemistry. *Chem. Eng. J.* **429**, 132548 (2022).
60 Huan, Y. *et al.* Achieving ultrahigh energy storage efficiency in local-composition gradient-structured ferroelectric ceramics. *Chem. Eng. J.* **425**, 129506 (2021).
61 Shi, C. *et al.* Significantly enhanced energy storage performances and power density in (1-x)BCZT-xSBT lead-free ceramics via synergistic optimization strategy. *Chem. Eng. J.* **426**, 130800 (2021).
62 Hu, D. *et al.* Optimization the energy density and efficiency of $BaTiO_3$-based ceramics for capacitor applications. *Chem. Eng. J.* **409**, 127375 (2021).
63 Cai, Z. *et al.* Local heterogeneous polarization enhanced superior low-field energy storage performance in lead-free relaxor ferroelectric ceramics. *ACS Sustain. Chem. Eng.* **11**, 13729-13735 (2023).
64 Qiao, X. *et al.* Superior comprehensive energy storage properties in $Bi_{0.5}Na_{0.5}TiO_3$-based relaxor ferroelectric ceramics. *Chem. Eng. J.* **388**, 124158 (2020).
65 Dong, X., Li, X., Chen, X., Wu, J. & Zhou, H. Simultaneous enhancement of polarization and breakdown strength in lead-free $BaTiO_3$-based ceramics. *Chem. Eng. J.* **409**, 128231 (2021).
66 Hu, Q. *et al.* Achieve ultrahigh energy storage performance in $BaTiO_3$-$Bi(Mg_{1/2}Ti_{1/2})O_3$ relaxor ferroelectric ceramics via nano-scale polarization mismatch and reconstruction. *Nano Energy* **67**, 104264 (2020).
67 Zhang, X. *et al.* Ultrahigh energy storage with superfast charge-discharge capability achieved in linear dielectric ceramic. *J. Mater. Sci. Technol.* **177**, 59-67 (2024).
68 Wei, T. *et al.* Novel $NaNbO_3$-$Sr_{0.7}Bi_{0.2}TiO_3$ lead-free dielectric ceramics with excellent energy storage properties. *Ceram. Int.* **47**, 3713-3719 (2021).
69 Yang, H. *et al.* Novel $BaTiO_3$-based, Ag/Pd-compatible lead-free relaxors with superior energy storage performance. *ACS Appl. Mater. Interfaces* **12**, 43942-43949 (2020).
70 Zhang, L. *et al.* Stronger B-site ionic disorder boosting enhanced dielectric energy-

storage performance in BNT-based relaxor ferroelectric ceramics. *Ceram. Int.* **49**, 7905-7912 (2023).
71 Li, D. *et al.* Enhanced energy storage properties achieved in $Na_{0.5}Bi_{0.5}TiO_3$-based ceramics via composition design and domain engineering. *Chem. Eng. J.* **419**, 129601 (2021).
72 Niu, Z. *et al.* $Bi_{0.5}K_{0.5}TiO_3$-based lead-free relaxor ferroelectric with high energy storage performances via the grain size and bandgap engineering. *Mater. Today Chem.* **24**, 100898 (2022).
73 Zhao, W. *et al.* Broad-high operating temperature range and enhanced energy storage performances in lead-free ferroelectrics. *Nat. Commun.* **14**, 5725 (2023).
74 Zhang, X. *et al.* Improved energy-storage properties accompanied by reduced interfacial polarization in linear $Ca_{0.5}Sr_{0.5}TiO_3$ ceramic. *Ceram. Int.* **49**, 27589-27596 (2023).
75 Li, X. *et al.* Enhancement of energy storage and hardness of $(Na_{0.5}Bi_{0.5})_{0.7}Sr_{0.3}TiO_3$-based relaxor ferroelectrics via introducing $Ba(Mg_{1/3}Nb_{2/3})O_3$. *Chem. Eng. J.* **431**, 133441 (2022).
76 Yan, F. *et al.* Significantly enhanced energy storage density and efficiency of BNT-based perovskite ceramics via A-site defect engineering. *Energy Storage Mater.* **30**, 392-400 (2020).
77 Li, C. *et al.* Superior energy storage performance in $(Bi_{0.5}Na_{0.5})TiO_3$-based lead-free relaxor ferroelectrics for dielectric capacitor application via multiscale optimization design. *J. Mater. Chem. A* **10**, 9535-9546 (2022).
78 Zhou, X. *et al.* Optimized dielectric energy storage performance in ZnO-modified $Bi_{0.5}Na_{0.5}TiO_3$-$Sr_{0.7}Bi_{0.2}\square_{0.1}TiO_3$ ceramics with composite structure and element segregation. *Chem. Eng. J.* **458**, 141449 (2023).
79 Liu, J. *et al.* A synergistic two-step optimization design enables high capacitive energy storage in lead-free $Sr_{0.7}Bi_{0.2}TiO_3$-based relaxor ferroelectric ceramics. *J. Mater. Chem. A* **11**, 609-620 (2023).
80 Luo, C. *et al.* Significantly enhanced energy-storage properties of $Bi_{0.47}Na_{0.47}Ba_{0.06}TiO_3$-$CaHfO_3$ ceramics by introducing $Sr_{0.7}Bi_{0.2}TiO_3$ for pulse capacitor application. *Chem. Eng. J.* **429**, 132165 (2022).
81 Luo, N. *et al.* Constructing phase boundary in $AgNbO_3$ antiferroelectrics: pathway simultaneously achieving high energy density and efficiency. *Nat. Commun.* **11**, 4824 (2020).
82 Yan, F. *et al.* Superior energy storage properties and excellent stability achieved in environment-friendly ferroelectrics via composition design strategy. *Nano Energy* **75**, 105012 (2020).
83 Li, D. *et al.* Improved energy storage properties achieved in $(K,Na)NbO_3$-based relaxor ferroelectric ceramics via a combinatorial optimization strategy. *Adv. Funct. Mater.* **32**, 2111776 (2022).
84 Yan, F. *et al.* Boosting energy storage performance of lead-free ceramics via layered structure optimization strategy. *Small* **18**, 2202575 (2022).
85 Wang, H. *et al.* Hierarchically polar structures induced superb energy storage properties for relaxor $Bi_{0.5}Na_{0.5}TiO_3$-based ceramics. *Chem. Eng. J.* **471**, 144446 (2023).
86 Ji, H. *et al.* Ultrahigh energy density in short-range tilted NBT-based lead-free multilayer ceramic capacitors by nanodomain percolation. *Energy Storage Mater.* **38**,

113-120 (2021).
87 Zhang, L. *et al.* Achieving ultrahigh energy density and ultrahigh efficiency simultaneously via characteristic regulation of polar nanoregions. *Chem. Eng. J.* **465**, 142862 (2023).
88 Chen, H. *et al.* Excellent energy storage properties and stability of $NaNbO_3$-$Bi(Mg_{0.5}Ta_{0.5})O_3$ ceramics by introducing $(Bi_{0.5}Na_{0.5})_{0.7}Sr_{0.3}TiO_3$. *J. Mater. Chem. A* **9**, 4789-4799 (2021).
89 Qi, H., Xie, A., Tian, A. & Zuo, R. Superior energy-storage capacitors with simultaneously giant energy density and efficiency using nanodomain engineered $BiFeO_3$-$BaTiO_3$-$NaNbO_3$ lead-free bulk ferroelectrics. *Adv. Energy Mater.* **10**, 1903338 (2020).
90 Yan, F. *et al.* Composition and structure optimized $BiFeO_3$-$SrTiO_3$ lead-free ceramics with ultrahigh energy storage performance. *Small* **18**, 2106515 (2022).
91 Zhang, L. *et al.* Enhanced antiferroelectric-like relaxor ferroelectric characteristic boosting energy storage performance of $(Bi_{0.5}Na_{0.5})TiO_3$-based ceramics via defect engineering. *J. Materiomics* **8**, 527-536 (2022).
92 Sun, Z. *et al.* Superior capacitive energy-storage performance in Pb-free relaxors with a simple chemical composition. *J. Am. Chem. Soc.* **145**, 6194-6202 (2023).
93 Liu, H. *et al.* Chemical design of Pb-free relaxors for giant capacitive energy storage. *J. Am. Chem. Soc.* **145**, 11764-11772 (2023).
94 Guan, Z.-N. *et al.* Significantly enhanced energy storage performance of lead-free BiFeO3-based ceramics via synergic optimization strategy. *ACS Appl. Mater. Interfaces* **14**, 44539-44549 (2022).
95 Wu, S. *et al.* Superb energy storage capability for $NaNbO_3$-based ceramics featuring labyrinthine submicro-domains with clustered lattice distortions. *Small* **19**, 2303915 (2023).
96 Chen, L. *et al.* Outstanding energy storage performance in high-hardness $(Bi_{0.5}K_{0.5})TiO_3$-based lead-free relaxors via multi-scale synergistic design. *Adv. Funct. Mater.* **32**, 2110478 (2022).
97 Cao, W. *et al.* Interfacial polarization restriction for ultrahigh energy-storage density in lead-free ceramics. *Adv. Funct. Mater.* **33**, 2301027 (2023).
98 Xie, A. *et al.* $NaNbO_3$-$(Bi_{0.5}Li_{0.5})TiO_3$ lead-free relaxor ferroelectric capacitors with superior energy-storage performances via multiple synergistic design. *Adv. Energy Mater.* **11**, 2101378 (2021).
99 Wei, K. *et al.* Enhancing comprehensive energy storage properties in Pb-free relaxor AFE/FE system via heterogeneous structure tuning and defect engineering. *Acta Mater.* **278**, 120278 (2024).
100 Xie, A. *et al.* Supercritical relaxor nanograined ferroelectrics for ultrahigh-energy-storage capacitors. *Adv. Mater.* **34**, 2204356 (2022).
101 Cui, T. *et al.* Outstanding comprehensive energy storage performance in lead-free BiFeO3-based relaxor ferroelectric ceramics by multiple optimization design. *Acta Mater.* **240**, 118286 (2022).
102 Duan, J. *et al.* High-entropy superparaelectrics with locally diverse ferroic distortion for high-capacitive energy storage. *Nat. Commun.* **15**, 6754 (2024).
103 Luo, H. *et al.* Outstanding energy-storage density together with efficiency of above 90%

via local structure design. *J. Am. Chem. Soc.* **146**, 460-467 (2023).
104 Wei, T. *et al.* High-entropy assisted capacitive energy storage in relaxor ferroelectrics by chemical short-range order. *Nat. Commun.* **16**, 807 (2025).
105 Liu, H. *et al.* Chemical framework to design linear-like relaxors toward capacitive energy storage. *J. Am. Chem. Soc.* **146**, 3498-3507 (2024).
